# Matrix-Dependent Stability and Antibacterial Efficacy of Silver Nanoparticles: A Comparative Study of Anionic Carbopol vs. Non-Ionic Pluronic Hydrogels

**DOI:** 10.3390/pharmaceutics18030314

**Published:** 2026-03-01

**Authors:** Amane A. Alaroud, Suhad Bani Melhim, Fahmy Banat, Arshiya Husaini, Suha M. Abudoleh, Mahmoud Y. Alkawareek, Alaaldin M. Alkilany

**Affiliations:** 1 Department of Pharmaceutics and Pharmaceutical Biotechnology, Faculty of Pharmacy, Mu’tah University, Karak 61710, Jordan; a.alaroud@mutah.edu.jo; 2 Faculty of Pharmacy, Isra University, Amman 11622, Jordan; 3 Department of Clinical Pharmacy and Pharmacy Practice, Faculty of Pharmaceutical Sciences, The Hashemite University, Zarqa 13133, Jordan; 4 R&D Department, TQ Pharma, Amman 11512, Jordan; 5 Department of Pharmaceutical Sciences, College of Pharmacy, QU Health, Qatar University, Doha 2713, Qatar; 6 Department of Basic Pharmaceutical Sciences, Faculty of Pharmacy, Middle East University, Amman 11831, Jordan; 7 Department of Pharmaceutics and Pharmaceutical Technology, School of Pharmacy, The University of Jordan, Amman 11942, Jordan

**Keywords:** silver nanoparticles, anionic vs. non-ionic matrices, carbopol 934, pluronic F127, antibacterial activity, colloidal stability, aggregation, size-dependent efficacy, wound healing

## Abstract

**Background/Objectives:** Hydrogels infused with silver nanoparticles (AgNPs) are widely used for their antibacterial properties, yet their stability, specifically upon contact with solid growth media (agar), remains poorly explored. This study compared two hydrogel matrices, anionic Carbopol 934 and non-ionic Pluronic F127, incorporating AgNPs of three different sizes. The evaluation focused on colloidal stability and antibacterial efficacy against Gram-positive and Gram-negative bacteria. **Methods:** In this study AgNPs (~20, ~55, and ~65 nm) were synthesised via a wet-chemical method and characterised by UV–visible spectroscopy, dynamic light scattering (DLS), and transmission electron microscopy (TEM). AgNPs were incorporated into Carbopol 934 and Pluronic F127 hydrogel matrices. Colloidal stability was monitored over four months of storage and upon contact with tryptic soy agar (TSA). Antibacterial activity was assessed using agar diffusion assays. **Results:** Showed that both hydrogel systems maintained AgNP stability during storage, comparable to aqueous suspensions. However, upon contact with TSA, aggregation of Carbopol–AgNP hydrogels occurred, whereas Pluronic–AgNP hydrogels remained stable. In antibacterial assays, both hydrogels produced larger zones of inhibition (ZOI) than AgNP suspensions against Gram-negative bacteria (*E. coli*, *P. aeruginosa*), with Carbopol–AgNP hydrogels demonstrating superior efficacy in an inverse size-dependent manner. Against Gram-positive bacteria (*S. aureus*, *S. epidermidis*), Pluronic–AgNP hydrogels initially showed larger ZOIs due to the polymer’s intrinsic antibacterial activity. However, after correcting for this baseline, Carbopol–AgNP hydrogels exhibited superior net efficacy, with *S. epidermidis* showing greater susceptibility than *S. aureus*. **Conclusions:** While both Carbopol 934 and Pluronic F127 stabilise AgNPs during storage, the matrix type significantly influences behaviour at the biological interface. Carbopol–AgNP hydrogels aggregate upon contact with solid agar yet deliver superior, size-dependent antibacterial activity compared to the stable but less potent Pluronic systems.

## 1. Introduction

Silver nanoparticles (AgNPs) represent a significant development in nanotechnology, particularly in fighting against multidrug-resistant pathogens. They have been investigated as broad-spectrum antibacterial and antifungal agents [1,2]. Mechanisms of action include microbial membrane disruption, generation of reactive oxygen species (ROS), release of Ag^+^ and interfering with intracellular species [3,4]. Given that the use of AgNP suspensions in biomedical applications is limited by colloidal instability, aggregation, and uncontrolled release—which may compromise their efficacy—such suspensions also fail to adequately reflect the performance of pharmaceutical dosage forms [5,6]. Incorporating AgNPs within hydrogels has become the favoured choice for topical delivery systems, as they offer a hydrated, biocompatible matrix that stabilises nanoparticles, allows a controlled release of Ag^+^ ions and localises delivery at infection sites. They also offer a simple applicability with excellent spreadability properties, enabling extended local-delivery duration at the wound or infected sites [7,8,9,10].

Hydrogels are extensively utilised in topical and localised dosage forms, serving both as inert carriers and as active modulators of drug release, stability, and therapeutic efficacy. The influence of hydrogel matrix composition on formulation performance has been studied for various pharmaceutical ingredients, demonstrating its effects on the physicochemical stability, release behaviour, and therapeutic performance of incorporated agents. Several marketed formulations, such as SilvrSTAT^®^ and Nano-Silver Gel^®^, composed of 32 ppm AgNPs [11,12], Silvex^®^, and SilverFern NanoGel^®^, containing 24 ppm infused in carbomer hydrogel [13,14], demonstrate the clinical potential of AgNP-infused hydrogels. Results from a randomised controlled study using SilvrSTAT Gel in nonischaemic diabetic foot ulcers reported accelerated healing with 90% of ulcers fully healed by week 12, compared to 75% in the control group [15].

The hydrogels used for nanoparticle delivery or for other topical biomedical applications can be broadly classified based on the nature of the charge of the constituent polymer as ionic or non-ionic hydrogels [16,17]. This classification of hydrogels is of significant importance for nanoparticle stabilisation in the hydrogel matrix. In ionic hydrogels, particularly anionic hydrogels based on poly(acrylic acid), stabilisation of nanoparticles occurs by electrostatic interactions between the nanoparticle surfaces and charged functional groups [18]. In contrast, non-ionic hydrogels are mainly driven by steric stabilisation effects, where the polymer chain creates a barrier that limits nanoparticle–nanoparticle interactions with minimal electrostatic effects [18]. The steric stabilisation effects are typical for amphiphilic block copolymer, where the hydrated polymer chain creates a corona that shields the nanoparticles from interaction [19]. The unique differences in stabilisation effects are recognised to affect the physicochemical stability of nanoparticles, interfacial interactions with living systems, and antimicrobial effects, emphasising the need for rational classification of hydrogels.

For example, ibuprofen incorporated into different hydrogel polymers (xanthan gum, sodium carboxymethylcellulose, poloxamer 407, and carbomer) exhibited distinct stabilities, release behaviours and suitabilities for skin application. The polymer type significantly affected the shape, size and degree of drug crystallisation, as well as the release kinetic parameters [20]. Another study reported that Carbopol gels influence the release kinetics of the weakly basic drug timolol via pH-dependent ionisation and polymer–drug complexation, resulting in slower and more regulated diffusion and sustained release than standard solutions [21]. Similarly, temperature-sensitive Pluronic F127-based hydrogels improve the solubility and stability of the poorly soluble curcumin, enabling sustained release of the medicinal agent, extended delivery duration, and preserved biological activity [18,22]. This underscores that the physicochemical characteristic of the hydrogel matrix dictates drug release and formulation stability

Similarly, hydrogels have been shown to modulate nanomaterial behaviour. AgNPs have been infused in various hydrogel formulations, including Carbopol [23,24,25], Pluronic [26,27], poly(vinyl alcohol) (PVA) [28], alginate lignin-based hydrogels [29], and chitosan–polyethylene glycol (PEG) hydrogels [30]—all have demonstrated antibacterial effects and wound healing outcomes, showing reduced inflammation and accelerated wound healing. Additionally, incorporating AgNPs in a crosslinked hydrogel composed of konjac glucomannan and chitosan was shown to reduce toxicity compared to AgNP suspension [31]. Although the therapeutic and antibacterial effectiveness of AgNPs infused in different hydrogel matrices has been studied, however, not enough has been done to compare the various hydrogel matrix types in order to provide the most efficient and stable environment for AgNP delivery [32]. Moreover, several studies have examined the stability of AgNPs in suspension in various biological growth media [5,33]; however, no studies have investigated how gel matrix type may influence the colloidal stability of AgNPs during long-term storage or upon exposure to growth media.

Among the factors affecting AgNP hydrogel performance are particle size and matrix charge. Smaller AgNPs (1–20 nm) generally show higher antibacterial activity [34,35,36,37,38], yet larger particles (30–100 nm) retain efficacy and may sustain inhibition longer under aggregation conditions [5,35,39]. Only a few studies have assessed the role of AgNP size in hydrogel systems; one report studied 10 nm and 50 nm AgNPs in alginate hydrogel [40], while another report investigated a narrower range of spherical AgNPs from 3 nm to 6.48 nm in PVA hydrogel [41]; however, none have investigated the effect of AgNP size on colloidal stability within hydrogels. Likewise, hydrogel matrix charge modulates AgNP behaviour. A previous study has reported that the bactericidal activity of AgNPs was significantly more effective for charged (ionic) hydrogel matrices compared to non-ionic matrices [42]. AgNPs have been incorporated into Carbopol hydrogels, an anionic matrix known to be an efficient matrix for AgNPs incorporation, demonstrating antibacterial and wound healing effects, and used in SilvrSTAT^®^ [23,24,25,43]. Furthermore, AgNPs infused into Pluronic^®^ F127 hydrogel, a non-ionic matrix, proved to be efficient for wound healing and antibacterial effects [26,44].

Therefore, this study aimed to systematically investigate the effect of hydrogel matrix charge and nanoparticle size on the colloidal stability over time and upon contact with agar growth media, as well as the effects on antibacterial efficacy of AgNP–hydrogel formulations. Carbopol was chosen as an example of an anionic hydrogel, typically used in pharmaceutical or wound healing applications, in which AgNP stabilisation is dominated by electrostatic interactions from carboxylate groups [45]. In contrast, Pluronic F127 was chosen as an example of a non-ionic hydrogel in which AgNP stabilisation is dominated by steric effects from the structure of the amphiphilic block copolymer [22]. Hence, these two hydrogels represent different AgNP stabilisation environments that operate by different mechanisms. The primary objective of this work was not to explore a variety of hydrogel formulations but to carry out a controlled comparison of two of the most used hydrogel types, ionic versus non-ionic hydrogels, to determine the effect of hydrogel charge and stabilisation mechanism on AgNP colloid stability, interfacial interactions, and antibacterial activity. 

## 2. Materials and Methods

### 2.1. Synthesis of Silver Nanoparticles with Various Sizes

Citrate-capped AgNPs of varying sizes were produced through a modified wet-chemical reduction approach, where silver nitrate (99.85%, analysis grade, Thermo Fisher Scientific, UK) was reduced using trisodium citrate (≥99.0%, American Chemical Society (ACS)-grade, Thermo Fisher Scientific, UK), and tannic acid (Merck, Darmstadt, Germany), as outlined by Bastús et al. The concentration of tannic acid (Ph. Eur./JP/USP, Merck, Darmstadt, Germany) was modified to regulate the size of the nanoparticles [46].

#### 2.1.1. Synthesis of ~20 nm Seed AgNPs

An aqueous solution of 100 mL was prepared, containing 5 mM trisodium citrate and 0.1 mM tannic acid, utilising Ultrapure water (18.2 MΩ·cm) obtained using a Milli-Q system (Merck Millipore, Burlington, MA, USA). The solution was placed in a three-neck round-bottom flask and heated to boiling while being stirred vigorously with an electro-heating mantle (Bibbby Scientific’s, Stone, Staffordshire, UK). A condenser was installed to inhibit the evaporation of the solvent. Upon reaching boiling, 1 mL of freshly prepared 25 mM AgNO_3_ (Thermo Fisher Scientific, Leicestershire, UK) solution was added dropwise through one neck of the flask, to produce a final concentration of 0.25 mM, leading to the immediate appearance of a yellow suspension, which signifies the formation of AgNPs. The purification of synthesised AgNPs was achieved through centrifugation with a HERMLE Z216M centrifuge (Marshall Scientific, Hampton, NH, USA) at 12,000 rpm for a duration of 20 min at room temperature.

The final pH of the reaction mixture, after the addition of AgNO_3_ and the formation of the nanoparticles, is around a neutral pH, between 6.8 and 7.0, owing to the buffering action of trisodium citrate and tannic acid.

#### 2.1.2. Seed-Mediated Growth of Larger AgNPs

The synthesis of larger silver nanoparticles (AgNPs) was carried out using the seed-growth method (Pacioni et al., 2015) [47], where 20 nm AgNPs were used as seeds. The solution was modified by removing 19.5 mL of the seed solution and adding 16.5 mL of Milli-Q water into the same solution, as described by Bastús et al. [46]. The temperature was decreased to 90 °C after boiling. At approximately 1 min intervals, 0.5 mL of 25 mM trisodium citrate, 1.5 mL of 2.5 mM tannic acid, and 1 mL of 25 mM AgNO_3_ were added sequentially. The reaction was carried out without any adjustments to the pH, as the pH level remained neutral, ranging from 6.8 to 7.0. In this type of growth system, tannic acid played a role as a growth regulator as well as a stabiliser, in addition to its mild reduction properties during the deposition of Ag^+^ onto the pre-formed seeds. The product of the initial growth phase (Generation 1, G1) was collected following a duration of 20 min at room temperature. Through a series of AgNO_3_ injections, which were both repeated and adjusted, multiple growth generations (G2, G3, etc.) were achieved until the target particle sizes were reached. The purification of synthesised AgNPs was achieved through centrifugation with a HERMLE Z216 centrifuge at 8000 rpm for 15 min. Prior to analysis, pellets were re-dispersed in a 2.2 mM trisodium citrate aqueous solution.

### 2.2. Apparatus

The prepared AgNPs suspensions were characterised using UV–visible spectroscopy (Evolution 300 UV-Vis Spectrophotometer, Thermo Fisher Scientific, Waltham, MA, USA). The morphology and size of AgNPs were assessed by transmission electron microscopy (TEM) using 200-mesh Formvar-coated copper grids (SPI Supplies, West Chester, PA, USA). A drop of the purified nanoparticle dispersion (10 nM) was placed onto the grid, excess liquid was carefully blotted with filter paper, and the grid was air-dried at room temperature overnight. Imaging was conducted using a Morgagni–Philips 268 FEI TEM equipped with a Mega-View G2 digital camera (Olympus Soft Imaging Solutions, Center Valley, PA, USA). Bright-field micrographs were collected at 80,000× to screen grid regions and evaluate dispersion quality, while 120,000× was used as the primary magnification for particle diameter measurements. When needed to confirm particle boundaries or assess subtle shape features, selected fields were additionally imaged at 200,000–300,000, with dynamic light scattering (DLS), and zeta potential analysis (Zetasizer Nano-ZS, Malvern Instruments, Malvern, Worcestershire, UK). Average diameter of each size of synthesised AgNPs was calculated from TEM images using ImageJ software (Version 1.54r). The concentration of silver nanoparticles was measured using UV–visible spectroscopy by utilising the maximum absorbance value related to the phenomenon of localised surface plasmon resonance (LSPR) and by using the reference data for the size-dependent extinction coefficients of citrate-capped silver nanoparticles to calculate the concentration using the Beer–Lambert law given by Paramelle et al. [48].

### 2.3. Evaluation of Colloidal Stability

The colloidal stability of purified AgNP suspensions was assessed under two conditions: (i) after being stored at room temperature in the dark (ageing) and (ii) after dilution with tryptic soy broth (TSB; EP/USP/ISO standards, CONDA Pronadisa, Madrid, Spain) at a 2:1 ratio (2 mL TSB: 1 mL of 1 mM AgNP suspension; this arbitrarily selected ratio was used to ensure a high concentration of AgNPs while keeping enough nutrition for growing bacteria). UV–visible spectra were obtained, and stability was evaluated by observing red shifts and the broadening of the LSPR peak, which indicates nanoparticle aggregation [49].

### 2.4. Incorporation of AgNPs in the Pharmaceutical Hydrogel Formulation

#### 2.4.1. Carbopol–AgNP Hydrogel Formulation

Carbopol^®^ 934 polymer is a white powder containing crosslinked polyacrylic acids. It is a carboxyl vinyl anionic polymer with a very high molecular weight.

It features short flow characteristics and a creamy flavour profile. The acid version of carbomer has 56–58% carboxylic acid groups, improving the polymer’s water solubility. When distributed in water, the carbomer molecules expand partially and create moderate viscosity. However, when neutralised with water-soluble alkali, the resin molecules swell entirely and form high viscosity [50].

A 20 mL aqueous solution with 32 ppm AgNPs (~20.1, 54.7, or 64.4 nm) was introduced into a mixer, and 0.25 g of Carbopol 934 powder (1.25% *w*/*v*) (pharmaceutical grade, United Pharmaceutical, Jordan) was slowly incorporated while maintaining continuous stirring until a uniform dispersion was obtained. The mixtures were permitted to hydrate for a minimum of 6 h, following which 1 N sodium hydroxide (NaOH; VWR BDH Prolabo, Leuven, Belgium) was introduced dropwise (approximately 14 drops per 20 mL) to achieve viscous hydrogels. Three more Carbopol hydrogels, each incorporating 32 ppm AgNO_3_, were created through adjustments of the total volume of NaOH used. All gels were kept in sealed, light-protected containers to avoid oxidative degradation of AgNPs [51].

#### 2.4.2. Poloxamer (Pluronic F127) Hydrogel Formulation

Pluronics^®^ are poloxamers made up of three blocks: polyethylene oxide (PEO), polypropylene oxide (PPO), and polyethylene oxide (PEO). Pluronics are the most extensively utilised steric stabilisers for formulations of nanostructured and/or nanosized drug delivery instruments due to their commercial availability and low cost. They induce micelle assembly and gelation at crucial micelle concentrations and temperatures [52]. A 20 mL aqueous solution containing 32 ppm AgNPs (~20.1, 54.7, or 64.4 nm) was introduced into a mixer, and 4 g of Pluronic F127 powder (20% *w*/*v*) (pharmaceutical grade, United Pharmaceutical, Amman, Jordan) was slowly incorporated while stirring. The solution was allowed to cool to 4 °C until full dissolution occurred [44].

#### 2.4.3. Characterisation of AgNP Hydrogels

UV–visible spectroscopy was used to monitor the optical properties and colloidal stability of AgNPs within the hydrogel matrices. All Carbopol 943- and Pluronic F127-based hydrogels were transparent and were analysed directly without dilution. Samples were placed in standard quartz cuvettes. Blank hydrogel samples, which were prepared using the same polymer compositions without the presence of AgNPs, were employed as control samples to account for the background absorption and any possible light scattering effects of the gel matrix. This allowed the effective monitoring of the LSPR band of the AgNPs, which could be indicative of any possible aggregation or dissolution of the particles. The viscosity was evaluated qualitatively with a spatula, and the pH was documented for each formulation. Samples of Pluronic F127 hydrogels were incubated at 37 °C before evaluation.

* No rheological evaluation was conducted and the consistency of the gel viscosity was controlled by using the same gelling agent (supplier, batch number) and final pH across all experiments.

#### 2.4.4. Evaluation of Colloidal Stability of AgNPs in Hydrogels

The assessment of colloidal stability was conducted following a 4-month storage period at room temperature in the absence of light. Hydrogel samples were transferred to cuvettes with a syringe to obtain UV–visible spectra. Stability was evaluated by observing variations in absorbance intensity and the position of the LSPR peak to identify any dissolution or aggregation of AgNPs.

Before conducting antibacterial tests, the colloidal stability in Tryptic Soy Agar (TSA; CONDA Pronadisa, Spain) was evaluated. Wells measuring 1.5 cm in diameter were created in TSA plates, and hydrogels containing 32 ppm were added to wells. The UV–visible spectra of the hydrogel–agar interface were obtained at intervals of 5 min, 60 min, 7 h, and 24 h to observe variations in the optical properties of AgNPs as a tool to evaluate their colloidal stability in gels.

### 2.5. Evaluation of Antibacterial Activity of AgNPs

#### 2.5.1. Bacterial Strains and Culture Conditions

The antibacterial efficacy of AgNP hydrogels was evaluated against Gram-negative bacteria *Escherichia coli* (ATCC^®^ 8739) and *Pseudomonas aeruginosa* (ATCC^®^ 47085), as well as Gram-positive bacteria *Staphylococcus aureus* (ATCC^®^ 29213) and *Staphylococcus epidermidis* (ATCC^®^ 12228). Tryptic soy broth and tryptic soy agar (CONDA Pronadisa, Spain) were used as growth medium. Long-term frozen stocks were created using TSB with 20% glycerol and maintained at −20 °C. Short-term cultures were preserved on streaked agar plates at 4 °C ( Colonies from overnight agar plates were suspended in sterile PBS and adjusted to the 0.5 McFarland standard for the experiments [53].

#### 2.5.2. Agar Diffusion (Cup–Plate) Assay for Hydrogels

The antibacterial assessment was conducted utilising hydrogels infused with either AgNPs or AgNO_3_, both maintained at a final concentration of 32 ppm, in conjunction with control hydrogels, employing the agar diffusion method. Moreover, suspensions of silver nanoparticles with varying particle sizes, along with an aqueous silver nitrate solution at a concentration of 32 ppm, were utilised as controls.

For each bacterial strain, 30 mL of molten TSA (approximately 40 °C) was inoculated with 20 µL of bacterial culture, mixed thoroughly using a vortex, and subsequently poured into sterile Petri dishes. Upon solidification, 6 mm wells were formed using a sterile cork borer, and the agar plugs were extracted aseptically. Each well was administered with 120 µL of the specified hydrogel. Plates were maintained at ambient temperature to allow gel diffusion, subsequently undergoing an overnight incubation at 32 °C. The diameters of the inhibition zones were quantified in triplicate [54].

## 3. Results

### 3.1. Characterisation of AgNPs

The synthesis of small-sized AgNPs was achieved through a wet-chemical method as described by Bastus et al., utilising the reduction of silver nitrate with trisodium citrate and tannic acid at elevated temperatures [46], while larger sizes were synthesised employing a seed-mediated protocol.

**Figure 1:**
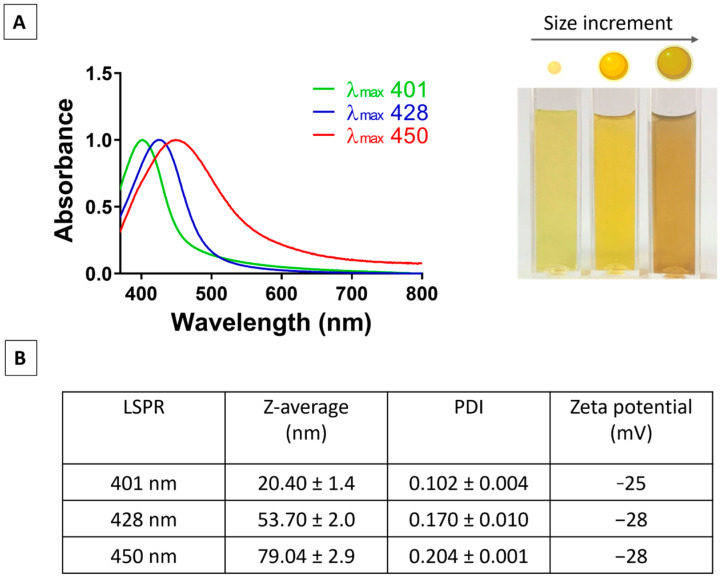
Characterisation of citrate-capped silver AgNPs. (**A**) UV–visible absorption spectra showing normalised LSPR peaks corresponding to increasing nanoparticle size, with a photograph for each size. (**B**) Summary table of LSPR peak positions, Z-average hydrodynamic diameters, polydispersity index (PDI), and zeta potential (mean ± SD, *n* = 3).

Figure 1A presents the UV–visible spectra of the prepared AgNP suspensions, showing LSPR peaks at 401, 428, and 450 nm, accompanied by vial images illustrating the corresponding colour changes with increasing particle size. Figure 1B provides the physicochemical characteristics of these AgNPs, with average hydrodynamic diameters of 20.4 nm (LSPR 401 nm), 53.7 nm (LSPR 428 nm), and 79.0 nm (LSPR 450 nm), together with their respective polydispersity index (PDI) and zeta potential values.

**Figure 2:**
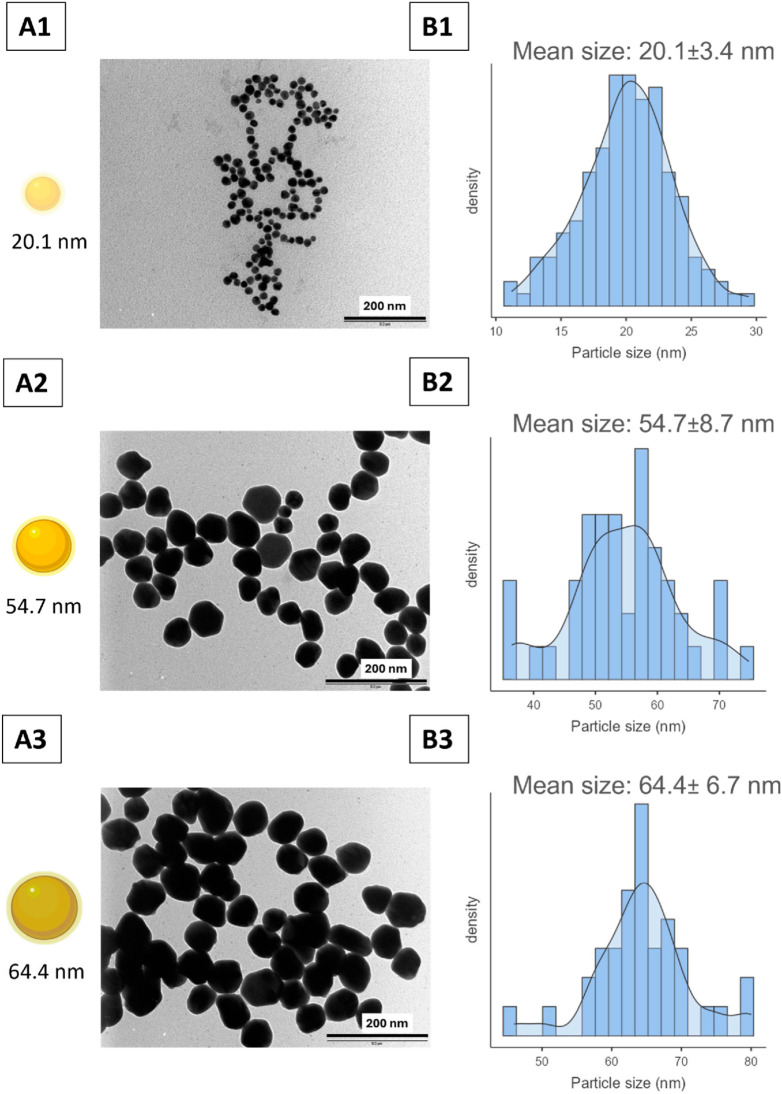
Representative TEM images of three synthesised citrate-capped AgNPs (**A1**–**A3**). Size distribution histograms (blue) with density curve (black line) of the synthesised AgNPs showing average diameter and standard deviation: (**B1**) 20.1 ± 3.4 nm (**B2**) 54.7 ± 8.7 nm (**B3**) 64.4 ± 6.7 nm. The average diameter and standard deviation for each size were calculated from TEM images using ImageJ software version 1.54r. Histograms were generated using Jamovi software version 2.6.44.

Figure 2(A1–A3) displays TEM images of the three synthesised particles along with analysis of TEM images, and the size distribution histograms are shown in Figure 2(B1–B3). The mean core nanoparticle size of the synthesised AgNPs was found to be 20.1 ± 3.4, 54.7 ± 8.7, and 64.4 ± 6.7 nm, respectively.

### 3.2. Colloidal and Chemical Stability of AgNP Suspensions upon Storage

**Figure 3:**
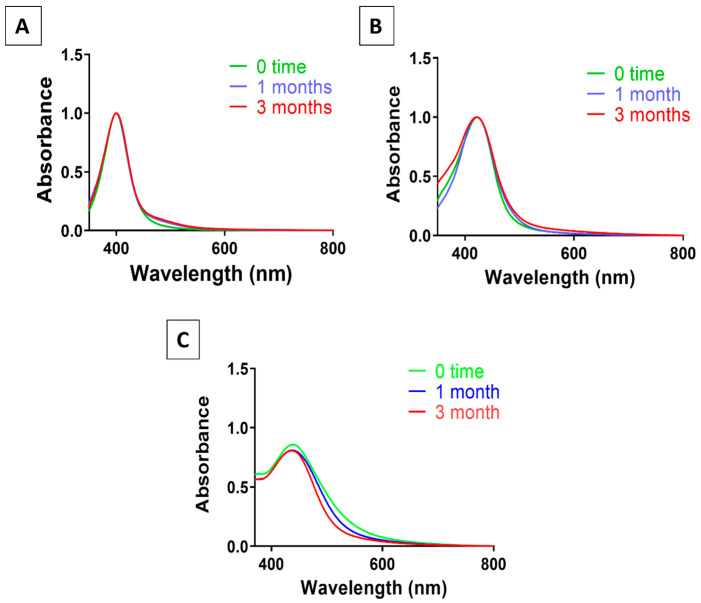
Colloidal stability of AgNP suspensions during storage. UV–visible spectra of (**A**) 20.1 nm, (**B**) 54.7 nm and (**C**) 64.4 nm AgNP suspension recorded immediately after preparation (0 time/months) and after storage at room temperature in dark conditions for up to three months.

Assessment of AgNP suspensions’ colloidal stability was performed after being stored for three months at room temperature for ~20.1 nm, 54.7 nm and 64.4 nm sizes for 3 months. AgNP suspensions demonstrated stability without aggregation or core dissolution, as indicated by their consistent UV-vis spectra compared to baseline (freshly made AgNP suspensions at 0 time), as illustrated in Figure 3.

### 3.3. Colloidal Stability of AgNP Suspensions upon Contact with Bacterial Growth Medium (TSB)

**Figure 4:**
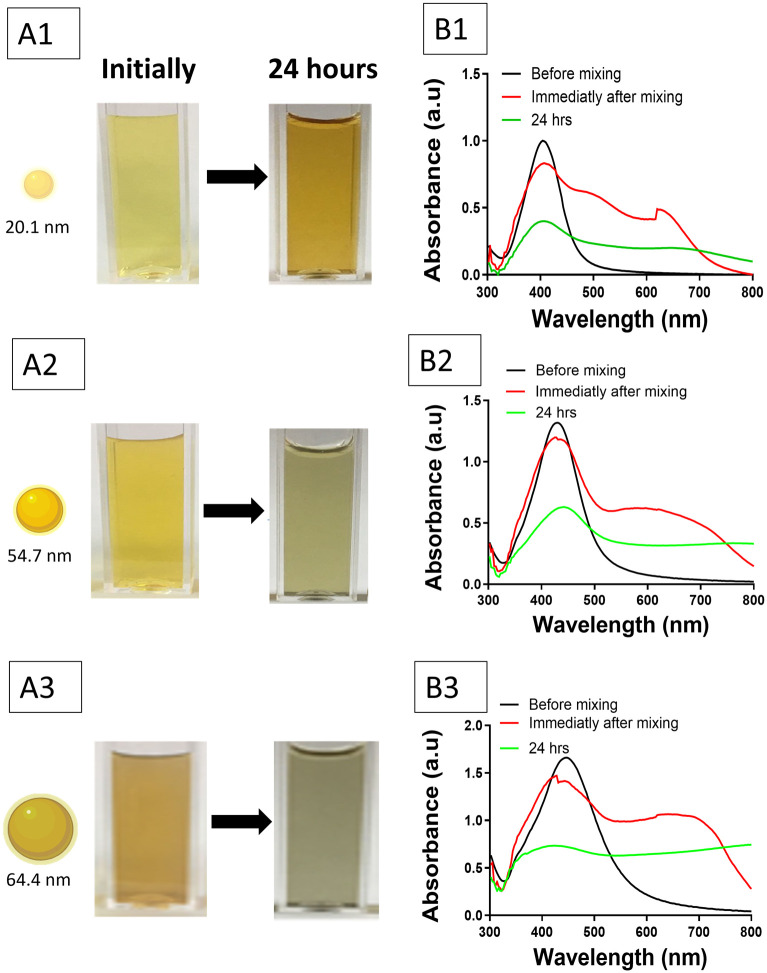
Colloidal stability of AgNP suspensions upon contact with bacterial growth medium (TSB). (**A1**–**A3**) Photographs of AgNP suspensions of three different sizes (20.1 nm, 54.7 nm and 64.4 nm) taken immediately and after 24 h of incubation at room temperature. (**B1**–**B3**) UV–visible spectra of AgNP suspensions of three different sizes (20.1, 54.7 and 64.4 nm) before mixing with TSB (black), immediately after mixing (red), and after 24 h of incubation at room temperature (green). Each row corresponds to a different AgNP size: (1) ~20.1 nm, (2) ~54.7, and (3) ~64.4 nm.

Moreover, stability testing of AgNP suspensions when combined with tryptic soy broth (TSB) was performed. Photographic evidence indicated a significant alteration in the colour of the solution, as the initially vibrant yellow suspensions gradually became less vivid over time. Immediate spectral alterations were noted, featuring a red shift and broadening of the LSPR peaks, which suggest aggregation of the nanoparticles. Within 24 h, there was an additional reduction in the LSPR peak, accompanied by a notable decline in absorbance intensity, as shown in Figure 4. For all sizes of AgNPs tested, an additional peak was observed at longer wavelengths (>500 nm) in the time intervals studied, compared to the initial spectra, indicating the aggregation of AgNP suspension. The observed effects were consistent across all tested sizes of AgNPs, indicating that extended exposure to TSB compromises the colloidal stability of AgNPs in suspension and encourages precipitation.

AgNP suspensions remained stable during long-term storage; however, the irreversible aggregation in bacterial growth media compromised their colloidal stability, making them unreliable for regular antibacterial evaluation. Accordingly, AgNPs were infused into Carbopol (anionic) and Pluronic F127 (non-ionic) hydrogel matrices to improve stability and ensure more accurate and reliable antibacterial testing.

### 3.4. Preparation of AgNP-Infused Hydrogels

The evaluation of hydrogels containing silver nanoparticles (AgNPs) included visual inspection, qualitative measurement of viscosity by spatula, pH quantification, and ultraviolet–visible spectroscopy to detect the possible dissolution of the cores of the nanoparticles and the aggregation of the particles. The hydration of the gelling polymers in suspensions containing the silver nanoparticles resulted in hydrogels with acceptable appearance and reproducibility of semi-solid consistency. Hydrogels containing Carbopol 943 had a firm, non-flowing consistency, while hydrogels containing Pluronic F127 had a softer, spreadable consistency. There were no discernible differences in qualitative viscosity and pH when compared to the control formulations.

**Figure 5:**
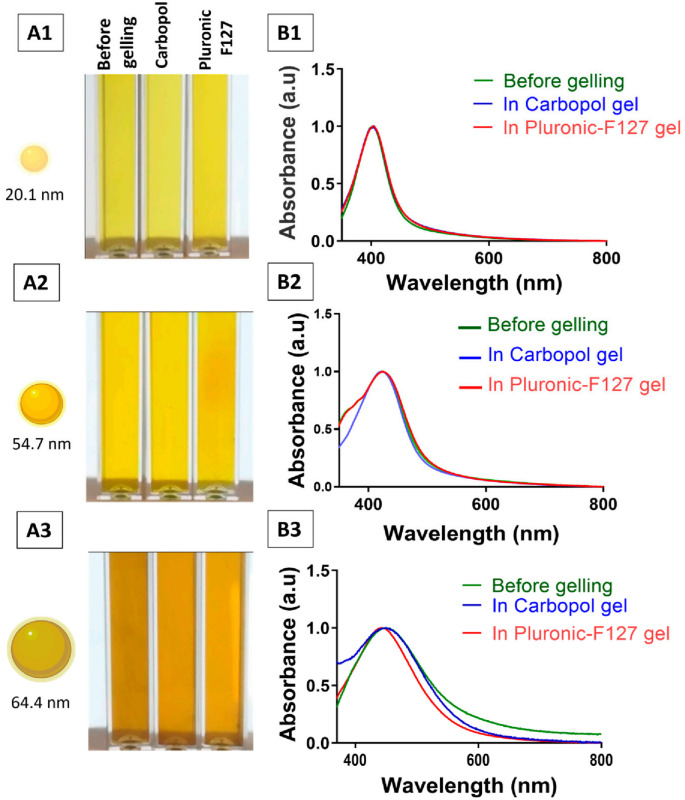
Colloidal stability of AgNPs during incorporation into either Carbopol or Pluronic F127 hydrogels. (**A1**–**A3**) Photographs of AgNP suspensions before gelling, after incorporation into Carbopol hydrogel and after incorporation into Pluronic F127 hydrogel. (**B1**–**B3**) Corresponding UV–visible spectra show no significant red shift or peak broadening of the LSPR after gel formation. Each row corresponds to a different AgNP size: (1) ~20.1 nm, (2) ~54.7, and (3) ~64.4 nm.

The pH of Carbopol–AgNP hydrogels ranged from 4.5 to 5.5, while Pluronic-based hydrogels showed slightly higher values between 5.5 and 6.0. The colour of the prepared AgNP hydrogels was consistent with that of the original nanoparticle suspension, indicating no visible aggregation during gel formation (Figure 5). UV–visible spectroscopy further confirmed colloidal stability, as no red shift or peak broadening of the localised surface plasmon resonance (LSPR) band was observed in AgNP hydrogels relative to their aqueous precursors (green line, before gelling).

### 3.5. Colloidal Stability of AgNPs in Hydrogels Throughout Storage

**Figure 6:**
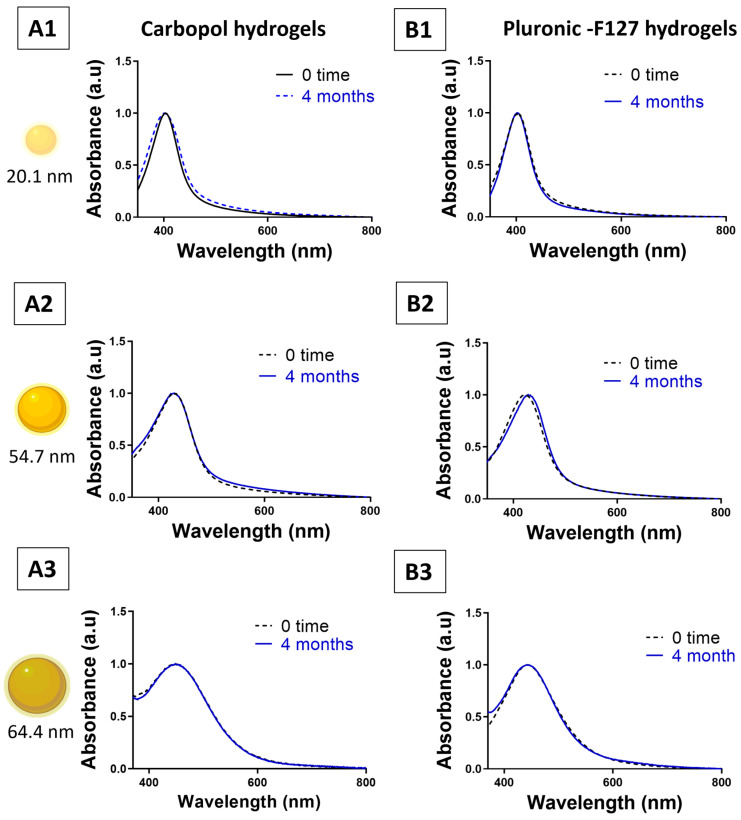
Colloidal stability of AgNP hydrogels during long-term storage. UV–visible spectra of Carbopol–AgNP hydrogels (**A1**–**A3**) and Pluronic–AgNP hydrogels (**B1**–**B3**) recorded immediately after formulation (0 time, Dark) and (after 4 months, blue) of storage at room temperature in the dark. Each row corresponds to a different AgNP size: (1) ~20.1 nm, (2) ~54.7, and (3) ~64.4 nm.

The UV–visible spectra of Carbopol–AgNP hydrogels and Pluronic F127–AgNP hydrogels of different sizes were recorded throughout the storage period. The LSPR peaks remained essentially superimposable with the initial spectra obtained at formulation (t = 0), showing complete overlap with no observable red shift or peak broadening (Figure 6). These findings confirm excellent colloidal stability of the incorporated AgNPs. Furthermore, the absorbance values remained consistent over the entire storage duration, indicating that AgNPs preserved their colloidal and chemical stability within both hydrogel matrices.

### 3.6. Colloidal Stability of AgNP Hydrogels in Bacterial Growth Media (TSA)

**Figure 7:**
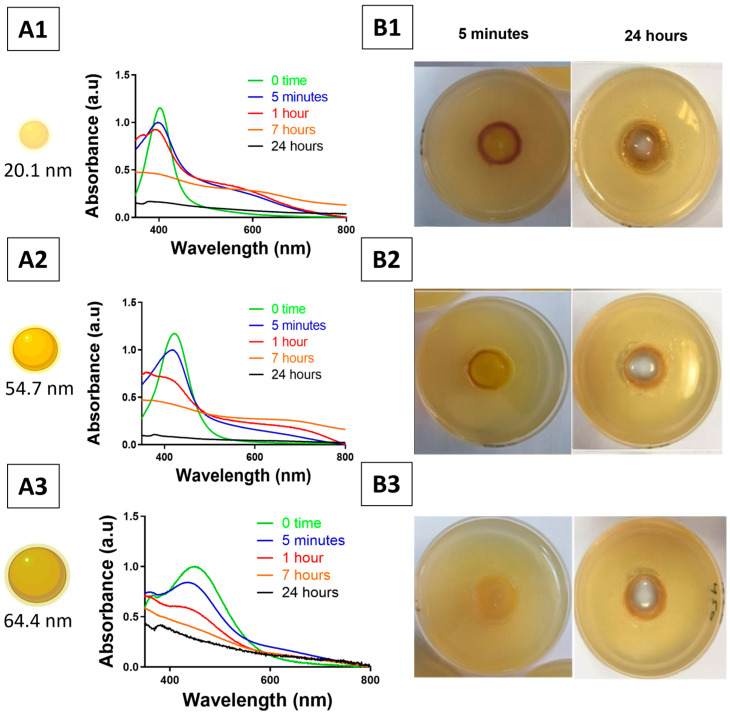
Colloidal stability of Carbopol–AgNP hydrogels upon contact with bacterial growth medium (TSA) over time. (**A1**–**A3**) UV–visible spectra of Carbopol–AgNP hydrogels recorded at 0 time, 5 min, 1 h, 7 h, and 24 h after placement in TSA wells. Progressive red shift, peak broadening, and decreased absorbance indicate nanoparticle aggregation and possible core dissolution. (**B1**–**B3**) Corresponding photographs of the TSA plates at 5 min and 24 h. The colourless area in the middle of each well corresponds to the original hydrogel plug (source area) and is not an air bubble. The coloured area surrounding this indicates the diffusion of AgNPs and/or silver into the agar gel. This has formed a diffusion zone that is observable. Each row corresponds to a different AgNP size: (1) ~20.1 nm, (2) ~54.7, and (3) ~64.4 nm.

The prolonged stability of AgNP suspensions was initially confirmed. Afterwards, the colloidal stability of Carbopol–AgNP hydrogels was evaluated following contact with tryptic soy agar (TSA) using visual inspection and UV–visible spectroscopy. In the plate assays, the colourless region represents the hydrogel source, while the coloured ring represents the diffusion of the AgNP-related species and/or the released silver ions into the agar matrix. Although the above images provide a visual representation of the phenomenon of diffusion, the aggregation behaviour of the nanoparticles and the colloidal stability are mainly inferred from the time-dependent changes in the UV–visible spectra. In agar wells, Carbopol–AgNP hydrogels demonstrated a gradual colour transition from yellow to dark brown. Furthermore, the UV–visible spectra indicated the emergence of an extra absorbance band at wavelengths > 500 nm during the early contact intervals (5 min, 1 h, and 7 h), indicative of nanoparticle aggregation (Figure 7). The intensity of this band progressively diminished and became undetectable after roughly 24 h. The extent and rate of colour alteration were dependent upon nanoparticle sizes. Hydrogels with around 20.1 nm AgNPs demonstrated an almost instantaneous colour shift, whilst those with approximately 54.7 nm particles had more gradual alterations.

**Figure 8:**
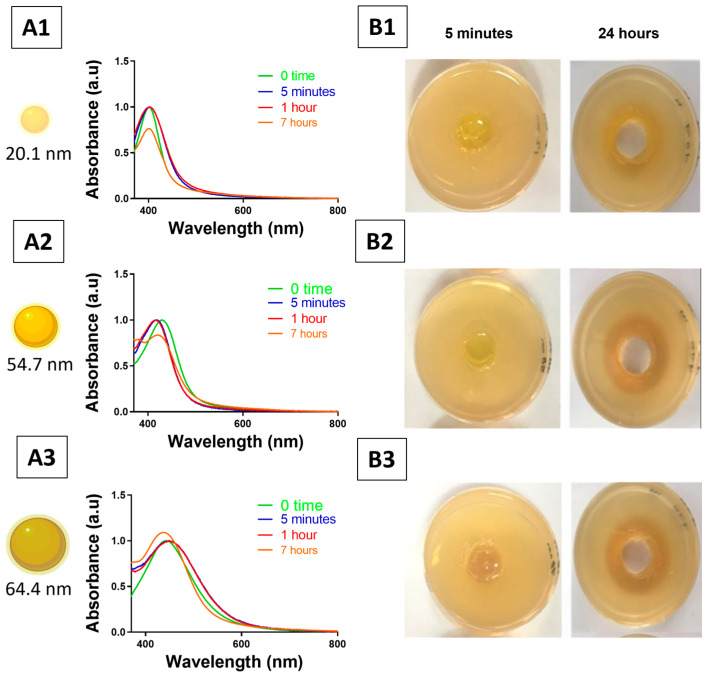
Colloidal stability and diffusion of Pluronic–AgNP hydrogel in tryptic soy agar (TSA). (**A1**–**A3**) UV–visible spectra of Pluronic–AgNP hydrogel of three sizes recorded at baseline (0 time), 5 min, 1 h, 5 h and 7 h after placement in TSA wells. (**B1**–**B3**) Corresponding photographs of TSA plates at 5 min and 24 h. The central colourless area in each well is the source of the hydrogel plug, indicating that there is no air bubble. The coloured ring around the hydrogel plug in each well shows the diffusion of AgNPs and/or silver, which creates a distinguishable optical-diffusion zone. Each row corresponds to a different AgNP size: (1) ~20.1 nm, (2) ~54.7, and (3) ~64.4 nm.

In contrast, the colour of Pluronic–AgNP hydrogel exhibited stability over time when placed in agar wells (Figure 8), demonstrating effective retention of nanoparticle stability within the polymer matrix during the initial contact period. For all AgNP sizes tested, no additional peak was observed at longer wavelengths (>500 nm) in all time intervals compared to 0 time, indicating the absence of AgNP aggregation in Pluronic F127 hydrogel formulation. The onset of AgNP diffusion into the agar was noted to commence around 7 h. A faster diffusion rate was observed for the ~20.1 nm size, whereas the larger particles (~54.7 and ~64.4 nm) exhibited a slower diffusion rate, as reflected by the smaller decrease in absorbance intensity. The introduction of Pluronic F127 hydrogels into agar wells led to a minor decrease in gel viscosity within the well. The UV–visible spectra of Pluronic–AgNP hydrogel in agar wells exhibited a subtle red shift only and minimal peak broadening of the LSPR, while absorbance progressively diminished alongside the diffusion of nanoparticles into the agar.

**Figure 9:**
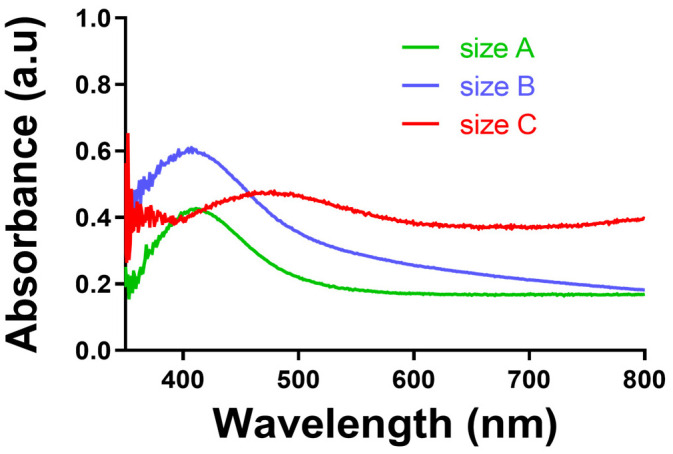
UV–visible spectra of excised agar following 24 h diffusion of Pluronic–AgNP hydrogels. Distinct LSPR peaks corresponding to AgNP sizes (A) ~20.1, (B) 54.7 and (C) 64.4 nm.

As shown in Figure 8, complete diffusion of Pluronic–AgNP hydrogels into the agar medium was observed after 24 h. To confirm the presence of AgNPs within the agar, UV–visible spectra of the excised agar samples were recorded (Figure 9). The spectra revealed distinct LSPR peaks corresponding to the tested nanoparticle sizes (~20.1, 54.7, or 64.4 nm), demonstrating effective migration of AgNPs from the hydrogels into the surrounding agar matrix. The spectral features varied with particle size: smaller nanoparticles (~20.1 nm) produced sharper, well-defined peaks, whereas larger nanoparticles (~64.40 nm) exhibited broader signals. Variations in peak sharpness and broadening reflect size-dependent diffusion kinetics and potential aggregation behaviour within the agar medium.

### 3.7. Antibacterial Activity of AgNP Hydrogels—Agar Diffusion Assay

#### 3.7.1. Gram-Negative Bacteria (*Escherichia coli* and *Pseudomonas aeruginosa*)

The zone of inhibition (ZOI) diameters for Carbopol–AgNP hydrogels and Pluronic–AgNP hydrogels against *E. coli* and *P. aeruginosa* are presented in Table 1. Blank hydrogels showed no antibacterial properties against both Gram-negative bacteria, while AgNP hydrogels and AgNO_3_ demonstrated greater inhibition zones compared to AgNP suspensions. Carbopol–AgNP hydrogels demonstrated superior inhibitory effects compared to Pluronic–AgNP hydrogels. The antibacterial activity was size-dependent in both hydrogel matrices, with the most pronounced effect observed for 20.1 nm AgNP hydrogels. Notably, Pluronic–AgNP hydrogels of 64.4 nm size showed no inhibition against *P. aeruginosa*.

#### 3.7.2. Gram-Positive Bacteria (*Staphylococcus aureus* and *Staphylococcus epidermidis*)

Table 2 presents the inhibition data for *S. aureus* and *S. epidermidis*. Blank Carbopol hydrogels alone did not have inhibitory effects; however, AgNP hydrogels exhibited a size-dependent inhibition for both Gram-positive bacteria, with a better effect against *S. epidermidis*, which displayed a greater sensitivity to AgNP hydrogels compared to *S. aureus*, *E. coli* or *P. aeruginosa.*

Although Pluronic–AgNP hydrogels showed larger ZOI compared to Carbopol–AgNP hydrogels for the same AgNP size, this effect is mainly attributable to the blank Pluronic F127 hydrogel itself (~12.5 mm) rather than the incorporated AgNPs. By subtracting from blank, the ZOI for Pluronic–AgNP hydrogel was approximately 1.5–1.75 mm upon incorporation of AgNPs of all sizes, compared to 7–13 mm for Carbopol–AgNP hydrogels. Thus, Gram-positive bacteria were more susceptible to Carbopol–AgNP hydrogels, with *S. epidermidis* displaying a greater susceptibility among all studied species.

It can be concluded that Carbopol–AgNP hydrogels showed a superior inhibitory activity, in a size-dependent manner, against both Gram-positive and Gram-negative bacteria studied compared to Pluronic–AgNP hydrogel and AgNP suspensions of all sizes. Furthermore, Gram-negative bacteria demonstrated a greater sensitivity to variations in size in Carbopol–AgNP hydrogels.

## 4. Discussion

This study compared Carbopol (anionic) and Pluronic F127 (non-ionic) hydrogel matrices infused with three different sizes of AgNPs, focusing on stability during long-term storage and upon contact with bacterial growth media, as well as antibacterial activity against representative strains of both Gram-positive and Gram-negative bacteria.

The reason why these particular hydrogels were selected is that they are well-known and established pharmaceutical excipients, which are generally used in existing approved drug formulations. In addition, silver-based carbomer gel formulations have already been used in existing approved medical device products, such as wound hydrogels. For example, a silver-based wound hydrogel, known as SilvrSTAT, is a carbomer gel incorporating AgNps [15,43,55,56].

First, this investigation involved the synthesis of AgNPs at three specific sizes (~20.1, 54.7, or 64.4 nm), utilising a modified citrate–tannic acid reduction approach in conjunction with a seed-mediated growth technique. This methodology, derived from the work of Bastús et al., employs tannic acid to facilitate nucleation and trisodium citrate to enhance growth and provide electrostatic stabilisation [46]. Through the adjustment of tannic acid concentration, it is possible to modulate the kinetics of reduction and the distribution of particle sizes, given that tannic acid not only complexes with Ag^+^ ions but also affects their rate of reduction. Reduced levels of tannic acid enhance the speed of nucleation and lead to the creation of small, uniform nanoparticles. As the concentration of tannic acid increased from 0.025 mM to 0.1 mM, the size of the prepared nanoparticles increased, and the spectrum shifted towards longer wavelengths (red shift), showing increasing size. The solution also changed colour from bright yellow to light brown. At concentrations of tannic acid exceeding 0.1 mM, the spectra exhibited broadening, and the polydispersity index (PDI) values increased above 0.25, signifying the formation of polydisperse nanoparticles. This issue was mitigated by employing a seed-mediated protocol that regulates both the seed particle concentration and the total number of silver atoms during each growth phase, thereby preventing the emergence of new AgNP nuclei, resulting in the formation of monodisperse AgNPs of larger sizes.

The tannic acid may play different roles: co-reductant, complexing agent of Ag+, and surface-active stabiliser. As a result, the change in tannic acid concentration may control the nucleation/growth rate ratio by affecting the reduction kinetics and the amount of Ag+ through complexation, thus controlling the nucleation density and the growth of the particles. Within the range of tannic acid concentrations tested in this work with constant citrate concentrations, the higher concentrations of tannic acid corresponded to larger sizes of AgNPs [46,57].

### 4.1. Stability of AgNPs in Suspension

Citrate-capped AgNP suspensions maintain their characteristic yellow and unique LSPR peaks for prolonged durations, 3 months, without exhibiting any signs of peak red shift, broadening, or the development of secondary absorption bands at longer wavelengths. The lack of these spectral alterations suggests that there is no significant core dissolution, particle growth, or irreversible aggregation taking place during the ageing process.

The lasting stability of citrate-capped AgNP suspensions has been extensively recorded in previous research; citrate ions exhibit a strong adsorption onto the surface of nanoparticles, resulting in a negative surface charge that generates electrostatic repulsion among the particles, consequently preventing aggregation. The electrostatic barrier that provides stabilisation plays a crucial role in maintaining the physicochemical integrity of nanoparticles throughout the storage process. Additionally, numerous investigations have indicated this effect when they are stored under suitable conditions—specifically in dark, oxygen-restricted environments at moderate temperatures [33,58,59,60].

However, exposure of citrate-capped AgNP suspensions to TSB markedly compromises the colloidal stability. As shown in Section 3.3, citrate-capped AgNP suspensions undergo rapid aggregation and loss of colloidal stability upon contact with bacterial growth media, as evidenced by the red-shifting/broadening of the LSPR band (the emergence of additional bands >500 nm) and reduced absorbance intensity, all of which indicate nanoparticle aggregation and eventual precipitation. The principal factor contributing to this instability is the comparatively weak interaction observed between citrate ions and the surface of silver nanoparticles. As a result, citrate ions are easily replaced by competing ligands in TSB, such as electrolytes and amino-containing substances like amino acids or proteins. The observed displacement undermines electrostatic stabilisation, thereby facilitating the aggregation of nanoparticles [61,62].

Research showed that nanoparticles capped with citrate exhibit metastability and are susceptible to aggregation when subjected to environmental stresses, including significant dilution, variations in temperature, or exposure to elevated electrolyte concentrations, which can effectively screen surface charges and reduce electrostatic repulsion, facilitating the destabilisation of nanoparticles [62,63]. Unfortunately, the aggregation of citrate-capped AgNPs could not be reversed under standard conditions, even with the application of agitation. The process of aggregation can adversely affect antibacterial efficacy, as sedimentation restricts the mobility of nanoparticles, hindering their ability to induce antibacterial activity.

The findings presented in this study underscore the importance of implementing standardised stability assessments in biologically relevant environments. This is particularly highlighted by the work of Duval et al. (2019), Vazquez-Muñoz et al. (2020), and Gimenez-Ingalaturre et al. (2022), who collectively point out that the physicochemical transformations of AgNPs in testing media—most notably aggregation, dissolution, and protein interactions—have the potential to mask their actual antimicrobial effectiveness [64,65,66].

Accordingly, we assessed the antimicrobial properties of AgNPs in a clinically relevant pharmaceutical hydrogel and examined the impact of various gelling agents on both the colloidal stability and antimicrobial effectiveness of AgNPs of three distinct sizes.

### 4.2. Stability of AgNPs in Hydrogels

Two gelling agents, Pluronic F127 and Carbopol, were selected for this study; both polymers have non-toxic and biocompatible features, providing calming and bioadhesive effects that are advantageous for wound-site healing [44,67,68].

The UV-Vis spectroscopy method was used as the primary analytical tool for the evaluation of the colloidal stability and optical properties of silver nanoparticles (AgNPs) in different hydrogel matrices during storage and after exposure to biological growth media. The use of this method was necessary to address the research question of interest in this study, which was to evaluate the effect of the hydrogel matrix chemistry, as reflected by the use of the anionic hydrogel matrix Carbopol and the non-ionic hydrogel matrix Pluronic, on the stability, diffusion, and antibacterial efficacy of the silver nanoparticles.

The resulting AgNP-infused hydrogels had sustained long-term stability, a key property attributed to the structural features of the polymers and the high viscosity of the gel matrices. Carbopol’s anionic, polyacrylic acid structure provides stability via electrostatic stabilisation. Upon neutralisation, it generates carboxylate groups that are expected to interact with the AgNP’s surface, thereby providing electrostatic stabilisation [24,45,67]. However, stability is provided by steric stabilisation in Pluronic F127, a poly (ethylene oxide)-poly (propylene oxide)-poly (ethylene oxide) (PEO-PPO-PEO) triblock copolymer with a non-ionic structure. Due to its amphiphilic nature, the hydrophobic PPO segment can adhere to the surface of the nanoparticle, and the hydrophilic PEO chains extend outward, providing a repulsive force, thereby stabilising the nanoparticles. [22,44,68,69].

Additionally, the colloidal stability of AgNPs infused in Carbopol and Pluronic F127 hydrogels was assessed following exposure to tryptic soy agar (TSA). Aggregation of Carbopol–AgNP hydrogels was observed, with the effect being more pronounced for smaller nanoparticles, consistent with their higher total surface area and greater reactivity [5]. Aggregation of Carbopol–AgNP hydrogels in TSA was transient, as indicated by the disappearance of absorbance at longer wavelengths after 24 h, whereas AgNP suspensions in TSB retained an absorbance of ~0.5, reflecting irreversible aggregation. Unlike aqueous suspensions, Carbopol–AgNP hydrogels showed no visible sedimentation, thereby enabling the continued dissolution of AgNPs and supporting the sustained release of silver ions.

Recent investigations indicate that the stability of AgNPs infused into Carbopol hydrogels is significantly influenced by the characteristics of Ag–polymer interactions and the properties of the surrounding medium. The ionised carboxylate groups of Carbopol exhibit weak adsorption onto the surfaces of silver nanoparticles through mechanisms of electrostatic attraction or coordination. However, these interactions can be easily disrupted in complex environments due to the presence of competing anions, including chloride, phosphate, and various organic ligands found in culture or biological fluids [43]. The absence of these stabilising interactions facilitates the aggregation of nanoparticles and hastens the release of silver ions. Moreover, the structural integrity of the Carbopol network is significantly affected by pH and ionic strength. Environments rich in electrolytes compress the electrical double layer, which diminishes inter-chain repulsion. Simultaneously, variations in pH modify carboxylate ionisation, both of which contribute to partial gel collapse and a reduction in viscosity [70].

In contrast, Pluronic–AgNP hydrogels maintained their consistency upon contact with TSA, being unaffected by pH or electrolyte content, even after 24 h of incubation. Notably, AgNPs diffused more extensively into the agar matrix in Pluronic F127 hydrogels compared to Carbopol, likely due to the presence of well-dispersed individual nanoparticles rather than aggregates, which would otherwise restrict diffusion; the observed stabilisation can be attributed to the distinctive triblock architecture of Pluronic F127, which consists of hydrophilic polyethylene oxide (PEO) and hydrophobic polypropylene oxide (PPO) segments, thereby offering substantial steric stabilisation of nanoparticles. This behaviour is consistent with previous experimental findings indicating that gels based on Pluronic F127 infused with AgNPs maintained their dispersion and exhibited significant antibacterial and antibiofilm properties both in vitro and in vivo. This includes thermo-reversible poloxamer gels with AgNP-targeting biofilms, burn-model F127 hydrogels, and temperature-sensitive Pluronic F127/F68 AgNP gels. Additionally, research has demonstrated the effectiveness of Pluronic F127 as a stabiliser or coating for AgNPs to mitigate aggregation and maintain their activity [26,71,72]

Furthermore, a previous study by Gimenez-Ingalaturre et al. showed that for the incorporation of surfactants, Pluronic F127 acts as a surfactant because of its amphiphilic nature [73]; it was observed to alleviate the considerable aggregation of AgNPs, known to reduce antibacterial efficacy [72].

### 4.3. Antibacterial Activity and Size Effects

In this study, an agar diffusion assay was used for the assessment of AgNP hydrogel activity; the choice of the agar-based diffusion assay over liquid-phase minimum inhibitory concentration (MIC) was necessitated by the need to preserve the colloidal stability of the nanoparticles. The LSPR peak broadening in broth (Section 3.3) indicates that liquid assays would lead to a non-representative estimation of efficacy due to irreversible aggregation, whereas agar diffusion preserves the hydrogel’s structural integrity and mimics topical delivery

It is evident from the results of the agar diffusion assay that AgNP suspensions showed no bioactivity. Moreover, agar diffusion testing of AgNPs embedded in hydrogels, namely, Carbopol and Pluronic F127, showed promising antibacterial activity.

As discussed in Section 3.3, citrate-capped AgNP suspensions are known to rapidly aggregate upon exposure to bacterial growth media, leading to a loss of colloidal stability. It is suggested that this might compromise the antibacterial efficacy of AgNPs in two ways: (a) by reducing the diffusibility of nanoparticles across the agar interface, and (b) by reducing the available surface area of the nanoparticles. Since oxidation/dissolution reactions are surface-mediated, it is logical to assume that reducing the surface area of AgNPs would lead to a reduction in the rate of production of silver ions from the nanoparticle surface, which would, in turn, compromise the antibacterial efficacy of AgNPs. This is also supported by the fact that AgNP antibacterial efficacy is surface-mediated by oxidation/dissolution reactions, where Ag+ ions are released from the nanoparticle surface [74,75].

In contrast, as discussed in Section 3.6, the incorporation of AgNPs into hydrogel matrices provides a microenvironment that limits uncontrolled aggregation and sedimentation, thus maintaining a dispersed state at the site of application. It is assumed that the hydrogel matrix provides a more sustained release of Ag^+^ by limiting aggregation and sedimentation and maximising the surface area and contact with the medium, although the release of Ag^+^ was not quantitatively assessed in this investigation. This is apparently matrix-dependent, as evidenced by the different stabilisation and diffusion behaviour of Carbopol and Pluronic F127 at the agar interface and their corresponding antibacterial properties.

Additionally, the results of the agar diffusion tests of hydrogels indicated that the size of the nanoparticles, the bacterial species involved, and the specific hydrogel utilised all played significant roles in the efficacy of bacterial eradication. Carbopol–AgNP hydrogels exhibited a larger ZOI compared to Pluronic–AgNP hydrogel when tested against Gram-negative bacteria, specifically *E. coli* and *P. aeruginosa*. The observed activity was size-dependent, with decreased activity upon increased particle size, consistent with the prevailing notion that smaller AgNPs exhibit greater activity due to their enhanced surface-to-volume ratio and accelerated Ag+ flux [76,77].

While the ZOI diameter for Gram-positive bacteria, *S. aureus* and *S. epidermidis*, was larger in the case of Pluronic–AgNP hydrogels compared to Carbopol–AgNP hydrogels, this impact is primarily due to the blank Pluronic F127 hydrogel rather than the incorporated AgNPs. This observation aligns with existing knowledge regarding the synergistic interactions between F127 gels and AgNPs, which enhance antibiofilm and antibacterial efficacy [78]. Therefore, by excluding Pluronic F127 activity, Gram-positive bacteria were more susceptible to Carbopol–AgNP hydrogels, an effect that is primarily due to the infused AgNPs. Carbopol–AgNP hydrogels showed a superior inhibitory activity against both Gram-positive and Gram-negative bacteria compared to Pluronic–AgNP hydrogels and AgNP suspensions of all sizes. One explanation could be that Carbopol hydrogels stabilise AgNPs sufficiently to permit dissolution and release of silver ions upon contact with biological media, whereas Pluronic F127 hydrogel stabilises AgNPs to a degree that inhibits their dissolution and release, thereby diminishing antibacterial activity.

Research has also shown the antibacterial properties of AgNPs in Pluronic F127 and Carbopol hydrogels, indicating the significant role of both the concentration of AgNPs and the hydrogel in determining the antibacterial properties. A previous study by Chen et al. investigated the antibacterial effect of AgNPs embedded in a Pluronic F127/F68 hydrogel, using nanoparticles with a mean size of 78 ± 10 nm at 50 mg/L (≈50 ppm) [27]. In comparison, our formulation employed smaller nanoparticles (20, 54, and 64 nm) at a lower silver concentration (32 ppm ≈ 32 mg/L), allowing evaluation of size-dependent antibacterial activity at reduced silver content. For *E. coli*, Chen et al. reported a ZOI of 8.2 ± 0.4 mm, while our results ranged from 9.88 ± 0.25 mm (20 nm) to 6.5 ± 0.57 mm (64 nm). For *P. aeruginosa*, the reported ZOI was 9.2 ± 0.2 mm, compared to 9.5 ± 0.70 mm to 8 ± 1.41 mm in our study. In both Gram-negative organisms, the smallest nanoparticles (20 nm) achieved comparable or superior inhibition at a lower silver concentration, highlighting the advantage of size optimisation. For *S. aureus*, Chen et al. reported a ZOI of 10.7 ± 0.3 mm, whereas our Pluronic–AgNP hydrogel produced 13.75 ± 0.5 mm at 32 ppm. However, Chen et al. did not include a blank control, whereas our study demonstrated that Pluronic F127 possesses intrinsic antibacterial activity against Gram-positive bacteria [27].

Compared with a previously reported Carbopol 940 gel (AgNPs 21.7 ± 4.42 nm, 200 mg/L = 200 ppm), the Carbopol 934–AgNP hydrogels in this study (three different sizes: 20.1, 54, and 64 nm at 32 ppm) exhibited larger inhibition zones against *P. aeruginosa* (10–12.75 mm) compared to 9.1 ± 0.7 mm reported by Prusty and Parida [79]. For *S. aureus*, the inhibition zone for the smallest nanoparticles (20.1 nm) in our study was 9.75 ± 0.50 mm, slightly lower than the 11.3 ± 0.8 mm in the reference study, which is comparable when considering the lower AgNP concentration in our gels [79]. These results highlight that despite differences in polymer type, nanoparticle size, and silver content, Carbopol 934 gels maintain effective antibacterial activity, with smaller nanoparticles providing the greatest inhibition.

Additionally, small-sized Carbopol–AgNP hydrogels tend to aggregate faster than larger sizes, and their antibacterial activity was higher. This can be explained as follows: the aggregates of non-sedimented small-sized AgNPs still have a higher exposed surface area than larger sizes, even without aggregation. The relationship between the size of AgNPs and the antibacterial activity was evaluated in this study, and it was observed that smaller sizes had higher antibacterial activity than larger ones for both hydrogels, which is attributed to the high surface area of small sizes, and as a result, their dissolution rate is higher. But Gram-negative bacteria were more sensitive to the change in size than Gram-positive bacteria [5,80].

It is worth noting that blank Pluronic F127 hydrogel had antibacterial activity only against Gram-positive bacteria, which lack an outer membrane; this inherent activity is probably due to the hydrophobic PPO block in Pluronic F127, which penetrates into the exposed peptidoglycan layer of the Gram-positive bacteria, facilitating the interaction with the underlying cytoplasmic phospholipid membrane and thus causing membrane destabilisation, consistent with the previously reported activity of Pluronic F127 by Tănase MA et al. [81]. In contrast, blank Pluronic F127 hydrogel had no effect against Gram-negative bacteria, as they have an outer membrane made up of phospholipids, proteins and lipopolysaccharides, creating additional barriers and preventing access of charged or large molecules to the inner targets [81].

The results obtained from the agar diffusion assay demonstrate that the antibacterial effect observed within the hydrogels was influenced by a multifaceted interaction involving particle size, the polymer matrix, and the structural characteristics of the bacterial cell wall. Furthermore, Gram-negative bacteria demonstrated a heightened sensitivity to variations in size compared to Gram-positive bacteria, which likely indicates inherent differences in their cell envelope structures and permeability characteristics. The findings indicate that the antibacterial efficacy is not attributable exclusively to the chemistry of silver; instead, it arises from the combined influences of nanoparticle dimensions, bacterial characteristics, and the physicochemical properties of the hydrogel matrix.

### 4.4. Considerations for Formulation Development

In summary, it is crucial to optimise both the size of the nanoparticles and the composition of the hydrogel matrix to effectively target specific pathogens. Additionally, conducting stability tests in the relevant assay medium is vital for accurately assessing antibacterial effectiveness. The infusion of smaller AgNPs within Carbopol matrices could yield enhanced efficacy in both Gram-negative and Gram-positive bacteria, yet the effect of size was more pronounced in Gram-negative bacteria. Conversely, for Gram-positive pathogens, systems based on Pluronic F127 may deliver both inherent and silver-enhanced benefits. Subsequent investigations ought to establish uniformity in stability measurements (hydrodynamic size/PDI, Ag^+^ release), correlate these with inhibition metrics, and expand to in vivo infection models to connect laboratory assays with clinical applications.

### 4.5. Scope Limitation/Future Work

It is recognised that a complete characterisation of the microstructure and mechanical properties of hydrogel materials, such as their viscoelasticity, morphology, and mechanical strength, is important for wound dressing applications. Future studies will include such methods as scanning electron microscopy, rheological tests, and mechanical tests to extend the present study. In the present study, the consistency of the formulation was ensured by using the same grade and source of the polymer and the same conditions to prepare all the hydrogels. This minimised the differences in the physical properties of the gels used in the study.

Furthermore, although antibacterial activity was confirmed through agar diffusion assays, time-dependent antibacterial-plate imaging and quantitative measurement of the inhibition zones were not carried out in the current study. The testing of antibacterial activity after long storage will be done in the future as part of the general study of stability and shelf life.

Future studies using skin graft/ex vivo skin infection models or in vivo models will provide a more relevant comparison of different capping agents and gelling agents used to formulate AgNP hydrogel formulations and will provide a better understanding of the stability–release paradox and the effectiveness of AgNPs against a variety of microbes in infected skin. In addition, future cytotoxicity and skin irritation assessments will be assessed.

## 5. Conclusions

This investigation elucidates the interplay between size, polymer matrix, and biological media in influencing the functionality of AgNP hydrogels. Citrate-capped AgNPs exhibited stability in aqueous environments as well as within Carbopol and Pluronic F127 matrices for extended periods; however, their behaviour altered upon interaction with TSA. Significant aggregation of AgNPs, particularly for 20.1 nm AgNPs, was noted in Carbopol–AgNP hydrogels. Pluronic gels, conversely, inhibited particle migration and limited spectrum drift, aligning with the anticipated effects of steric stabilisation provided by the block copolymer network. The observed variations in stability resulted in distinct antibacterial profiles for various organisms and matrices. For instance, Carbopol–AgNP hydrogels demonstrated enhanced efficacy against Gram-negative bacteria, with increased activity observed at smaller sizes. Pluronic–AgNP hydrogels showed anti-Gram-negative activity while also having intrinsic activity against Gram-positive bacteria, effectively inhibiting bacterial growth within the matrix.

This study holds importance due to several key factors: first, it compares Carbopol–AgNP hydrogels and Pluronic–AgNP hydrogels in terms of colloidal stability within pharmaceutical hydrogel matrices, both before and during antimicrobial testing, addressing a gap in existing research; second, it assesses how the type of gelling polymer—specifically, anionic versus non-ionic—affects the antibacterial efficacy of AgNPs; finally, it clarifies the role of nanoparticle size and bacterial type in determining the antimicrobial performance of AgNPs in hydrogel formulations.

## Figures and Tables

**Table 1:** The zone of inhibition (ZOI) diameter of AgNPs in suspension and in two different hydrogel delivery systems against Gram-negative bacteria (mean ± SD; *n* = 3).

	Zone of Inhibition (ZOI) Diameter in Millimetres (mm)					
Agent Used	Gram-Negative Bacteria					
	*E. coli*			*P. aeruginosa*		
	Suspension	Carbopol Hydrogel	Pluronic F127 Hydrogel	Suspension	Carbopol Hydrogel	Pluronic F127 Hydrogel
**Blank**	^NA^	-^a^	-^a^	^NA^	-^a^	-^a^
**AgNO_3_**	16 ± 0.50	15 ± 0.82	14.33 ± 0.58	15.66 ± 0.58	14 ± 1.41	13.5 ± 0.70
**AgNPs (~20.1 nm)**	-^a^	11.75 ± 0.13	9.88 ± 0.25	-^a^	12.75 ± 1.06	9.5 ± 0.70
**AgNPs (~54.7 nm)**	-^a^	9.25 ± 0.47	7.5 ± 1.0	-^a^	11.5 ± 0.71	8 ± 1.41
**AgNPs (~64.4 nm)**	-^a^	8.5 ± 0.48	6.5 ± 0.57	-^a^	10 ± 0.0	-^a^

^NA^: Not Available, -^a^: No antibacterial activity reported.

**Table 2:** The zone of inhibition (ZOI) diameter in millimetres (mm) of AgNPs in suspension and in two different hydrogel delivery systems against Gram-positive bacteria (mean ± SD; *n* = 3).

	Zone of Inhibition (ZOI) Diameter in Millimetres (mm)					
Agent Used	Gram-Positive Bacteria					
	*S. aureus*			*S. epidermidis*		
	Suspension	Carbopol Hydrogel	Pluronic F127 Hydrogel	Suspension	Carbopol Hydrogel	Pluronic F127 Hydrogel
**Blank**	^NA^	-^a^	12.5 ± 0.70	^NA^	-^a^	12.5 ± 0.71
**AgNO_3_**	16 ± 0.0	15 ± 1.00	15 ± 1.00	15 ± 0.50	14 ± 1.41	14.5 ± 0.71
**AgNPs (~20.1 nm)**	-^a^	9.75 ± 0.50	13.75 ± 0.50	-^a^	13 ± 1.41	13.5 ± 0.71
**AgNPs (~54.7 nm)**	-^a^	9.25 ± 0.96	13.75 ± 0.50	-^a^	12.5 ± 0.70	13.5 ± 0.71
**AgNPs (~64.4 nm)**	-^a^	7.75 ± 1.25	13.25 ± 0.70	-^a^	12 ± 1.41	13.5 ± 0.71

^NA^: Not Available, -^a^: No antibacterial activity reported.

## Data Availability

Data presented in this study is contained within the article. Further inquiries can be directed to the corresponding author.

## Abbreviations

The following abbreviations are used in this manuscript:

AgNPs	Silver nanoparticles
DLS	Dynamic light scattering
*E. coli*	*Escherichia coli*
LSPR	Localised surface plasmon resonance
PDI	Polydispersity index
PEG	Polyethylene glycol
*P. aeruginosa*	*Pseudomonas aeruginosa*
PEO	Poly(ethylene oxide)
PPO	Poly(propylene oxide)
PVA	Poly(vinyl alcohol)
ROS	Reactive oxygen species
*S. aureus*	*Staphylococcus aureus*
*S. epidermidis*	*Staphylococcus epidermidis*
TSA	Tryptic soy agar
TSB	Tryptic soy broth
TEM	Transmission electron microscopy
UV–Vis	Ultraviolet–visible spectroscopy
ZOI	Zone of inhibition

## References

[1] Jangid H., Singh S., Kashyap P., Singh A., Kumar G. (2024). Advancing biomedical applications: An in-depth analysis of silver nanoparticles in antimicrobial, anticancer, and wound healing roles. Front. Pharmacol..

[2] Dube E., Okuthe G.E. (2025). Silver Nanoparticle-Based Antimicrobial Coatings: Sustainable Strategies for Microbial Contamination Control. Microbiol. Res..

[3] Rai M., Yadav A., Gade A. (2009). Silver nanoparticles as a new generation of antimicrobials. Biotechnol. Adv..

[4] Franci G., Falanga A., Galdiero S., Palomba L., Rai M., Morelli G., Galdiero M. (2015). Silver Nanoparticles as Potential Antibacterial Agents. Molecules.

[5] Bélteky P., Rónavári A., Zakupszky D., Boka E., Igaz N., Szerencsés B., Pfeiffer I., Vágvölgyi C., Kiricsi M., Kónya Z. (2021). Are Smaller Nanoparticles Always Better? Understanding the Biological Effect of Size-Dependent Silver Nanoparticle Aggregation Under Biorelevant Conditions. Int. J. Nanomed..

[6] Liao C., Li Y., Tjong S.C. (2019). Bactericidal and Cytotoxic Properties of Silver Nanoparticles. Int. J. Mol. Sci..

[7] Yudaev P., Mezhuev Y., Chistyakov E. (2022). Nanoparticle-Containing Wound Dressing: Antimicrobial and Healing Effects. Gels.

[8] Goulart D.B. (2022). The use of silver hydrogel in wound treatment as an alternative to reduce antibiotic-resistant pathogens. Res. Soc. Dev..

[9] Wahid F., Zhong C., Wang H.-S., Hu X.-H., Chu L.-Q. (2017). Recent Advances in Antimicrobial Hydrogels Containing Metal Ions and Metals/Metal Oxide Nanoparticles. Polymers.

[10] Nandhini J., Karthikeyan E., Elizabeth Rani E., Karthikha V.S., Sakthi Sanjana D., Jeevitha H., Rajeshkumar S., Venugopal V., Priyadharshan A. (2024). Advancing engineered approaches for sustainable wound regeneration and repair: Harnessing the potential of green synthesized silver nanoparticles. Eng. Regen..

[11] ABL Medical, LLC SilvrSTAT™ Antibacterial Wound Dressing Gel Package Insert. https://www.henryschein.com/us-en/images/medical/SilvrSTAT-Antibacterial-Wound-Dressing-Gel-Package-Insert.pdf.

[12] SilverCeuticals Nano-Silver Gel®-32ppm Nano Silver Solution. https://silverceuticals.com/products/nano-silver-gel?srsltid=AfmBOopUBJiLzEYrY_7glHojfcCz_Oy5ZeR1D4KLUhAdFBxJy8SEX8mX.

[13] Brand S.F. SilverFern NanoGel®-Purified Silver 24 PPM AG404-Frequency Charged. https://www.silverfernbrand.com/products/nano-gel?srsltid=AfmBOoozRx1h4Z4XbvAcV_9hRFNl18spW4_EWONV-ze10i0jj_Sj0QDX.

[14] Prepared B.S.G. Silvex^®^ Wound Gel. https://www.walgreens.com/store/c/be-smart-get-prepared-silvex-wound-gel/ID=300436828-product.

[15] Essa M.S., Ahmad K.S., Zayed M.E., Ibrahim S.G. (2023). Comparative Study Between Silver Nanoparticles Dressing (SilvrSTAT Gel) and Conventional Dressing in Diabetic Foot Ulcer Healing: A Prospective Randomized Study. Int. J. Low. Extrem. Wounds.

[16] Shariati F.S., Ebrahimi T., Shariati A., Maghsood M., Delashoub M. (2025). Hydrogels: Properties, classifications, characterizations, and biomedical applications. Clin. Res. Stud..

[17] Thang N.H., Chien T.B., Cuong D.X. (2023). Polymer-Based Hydrogels Applied in Drug Delivery: An Overview. Gels.

[18] Dannert C., Stokke B.T., Dias R.S. (2019). Nanoparticle-Hydrogel Composites: From Molecular Interactions to Macroscopic Behavior. Polymers.

[19] Jacob S., Kather F.S., Boddu S.H.S., Attimarad M., Nair A.B. (2025). Nanosuspension Innovations: Expanding Horizons in Drug Delivery Techniques. Pharmaceutics.

[20] Djekic L., Martinović M., Dobričić V., Čalija B., Medarević Đ., Primorac M. (2019). Comparison of the Effect of Bioadhesive Polymers on Stability and Drug Release Kinetics of Biocompatible Hydrogels for Topical Application of Ibuprofen. J. Pharm. Sci..

[21] Vijaya Rani K.R., Rajan S., Bhupathyraaj M., Priya R.K., Halligudi N., Al-Ghazali M.A., Sridhar S.B., Shareef J., Thomas S., Desai S.M. (2022). The Effect of Polymers on Drug Release Kinetics in Nanoemulsion In Situ Gel Formulation. Polymers.

[22] Lupu A., Bercea M., Avadanei M., Gradinaru L.M., Nita L.E., Gradinaru V.R. (2025). Temperature Sensitive Pluronic F127-Based Gels Incorporating Natural Therapeutic Agents. Macromol. Mater. Eng..

[23] Martínez-Higuera A., Rodríguez-Beas C., Villalobos-Noriega J.M.A., Arizmendi-Grijalva A., Ochoa-Sánchez C., Larios-Rodríguez E., Martínez-Soto J.M., Rodríguez-León E., Ibarra-Zazueta C., Mora-Monroy R. (2021). Hydrogel with silver nanoparticles synthesized by Mimosa tenuiflora for second-degree burns treatment. Sci. Rep..

[24] Chen C.-Y., Yin H., Chen X., Chen T.-H., Liu H.-M., Rao S.-S., Tan Y.-J., Qian Y.-X., Liu Y.-W., Hu X.-K. (2020). Ångstrom-scale silver particle–embedded carbomer gel promotes wound healing by inhibiting bacterial colonization and inflammation. Sci. Adv..

[25] Badhwar R., Mangla B., Neupane Y.R., Khanna K., Popli H. (2021). Quercetin loaded silver nanoparticles in hydrogel matrices for diabetic wound healing. Nanotechnology.

[26] Chen M., Yang Z., Wu H., Pan X., Xie X., Wu C. (2011). Antimicrobial activity and the mechanism of silver nanoparticle thermosensitive gel. Int. J. Nanomed..

[27] Chen M., Pan X., Wu H., Han K., Xie X., Wedge D.E., Repka M.A., Wu C. (2011). Preparation and anti-bacterial properties of a temperature-sensitive gel containing silver nanoparticles. Pharmazie.

[28] Sala K., Bańkosz M., Placek H., Sztrumpf J., Kędzierska M., Tyliszczak B. (2024). Eco-Friendly Synthesized Silver-Nanoparticle-Modified PVA/PEG Hydrogels. Proceedings.

[29] Yu J., Ran F., Li C., Hao Z., He H., Dai L., Wang J., Yang W. (2024). A Lignin Silver Nanoparticles/Polyvinyl Alcohol/Sodium Alginate Hybrid Hydrogel with Potent Mechanical Properties and Antibacterial Activity. Gels.

[30] Majie A., Saha R., Sarkar A., Bhowmik R., Karmakar S., Sharma V., Deokar K., Haque A.u., Tripathy S.S., Sarkar B. (2024). A novel chitosan–PEG hydrogel embedded with in situ silver nanoparticles of *Clerodendrum glandulosum* Lindl. extract: Evaluation of its in vivo diabetic wound healing properties using an image-guided machine learning model. Biomater. Sci..

[31] Jiang Y., Huang J., Wu X., Ren Y., Li Z., Ren J. (2020). Controlled release of silver ions from AgNPs using a hydrogel based on konjac glucomannan and chitosan for infected wounds. Int. J. Biol. Macromol..

[32] Pangli H., Vatanpour S., Hortamani S., Jalili R., Ghahary A. (2020). Incorporation of Silver Nanoparticles in Hydrogel Matrices for Controlling Wound Infection. J. Burn Care Res..

[33] Ferreira A.M., Vikulina A., Loughlin M., Volodkin D. (2023). How similar is the antibacterial activity of silver nanoparticles coated with different capping agents?. RSC Adv..

[34] Morones J.R., Elechiguerra J.L., Camacho A., Holt K., Kouri J.B., Ramírez J.T., Yacaman M.J. (2005). The bactericidal effect of silver nanoparticles. Nanotechnology.

[35] Agnihotri S., Mukherji S., Mukherji S. (2014). Size-controlled silver nanoparticles synthesized over the range 5–100 nm using the same protocol and their antibacterial efficacy. RSC Adv..

[36] Baker C., Pradhan A., Pakstis L., Pochan D.J., Shah S.I. (2005). Synthesis and antibacterial properties of silver nanoparticles. J. Nanosci. Nanotechnol..

[37] Tang S., Zheng J. (2018). Antibacterial Activity of Silver Nanoparticles: Structural Effects. Adv. Healthc. Mater..

[38] Laib I., Gheraissa N., Benaissa A., Benkhira L., Azzi M., Benaissa Y., Abdelaziz A.G., Tian F., Walsh M., Bechelany M. (2025). Tailoring innovative silver nanoparticles for modern medicine: The importance of size and shape control and functional modifications. Mater. Today Bio.

[39] Martínez-Castañón G.A., Niño-Martínez N., Martínez-Gutierrez F., Martínez-Mendoza J.R., Ruiz F. (2008). Synthesis and antibacterial activity of silver nanoparticles with different sizes. J. Nanoparticle Res..

[40] Mahasawat D.P., Mudtaleb S., Eaidprap P. (2019). The Influence of Silver Nanoparticle Sizes on Antibacterial Activity, Cytotoxicity and Genotoxicity of Alginate Hydrogel Beads Containing Silver Nanoparticles. Appl. Mech. Mater..

[41] Rodríguez Nuñez Y.A., Castro R.I., Arenas F.A., López-Cabaña Z.E., Carreño G., Carrasco-Sánchez V., Marican A., Villaseñor J., Vargas E., Santos L.S. (2019). Preparation of Hydrogel/Silver Nanohybrids Mediated by Tunable-Size Silver Nanoparticles for Potential Antibacterial Applications. Polymers.

[42] Monerris M., Broglia M.F., Yslas E.I., Barbero C.A., Rivarola C.R. (2019). Highly effective antimicrobial nanocomposites based on hydrogel matrix and silver nanoparticles: Long-lasting bactericidal and bacteriostatic effects. Soft Matter.

[43] Moreno Ruiz Y.P., de Almeida Campos L.A., Alves Agreles M.A., Galembeck A., Macário Ferro Cavalcanti I. (2023). Advanced Hydrogels Combined with Silver and Gold Nanoparticles against Antimicrobial Resistance. Antibiotics.

[44] Francisco P., Neves Amaral M., Neves A., Ferreira-Gonçalves T., Viana A.S., Catarino J., Faísca P., Simões S., Perdigão J., Charmier A.J. (2023). Pluronic^®^ F127 Hydrogel Containing Silver Nanoparticles in Skin Burn Regeneration: An Experimental Approach from Fundamental to Translational Research. Gels.

[45] Haddadine N., Chalal S., Abouzeid K., Bouslah N., Benaboura A., El-Shall M.S. (2018). Preparation and characterization of carbopol-silver nanocomposites for efficient antimicrobial applications. Polym. Adv. Technol..

[46] Bastús N.G., Merkoçi F., Piella J., Puntes V. (2014). Synthesis of Highly Monodisperse Citrate-Stabilized Silver Nanoparticles of up to 200 nm: Kinetic Control and Catalytic Properties. Chem. Mater..

[47] Pacioni N.L., Borsarelli C.D., Rey V., Veglia A.V., Alarcon E.I., Griffith M., Udekwu K.I. (2015). Synthetic Routes for the Preparation of Silver Nanoparticles. Silver Nanoparticle Applications: In the Fabrication and Design of Medical and Biosensing Devices.

[48] Paramelle D., Sadovoy A., Gorelik S., Free P., Hobley J., Fernig D.G. (2014). A rapid method to estimate the concentration of citrate capped silver nanoparticles from UV-visible light spectra. Analyst.

[49] Carlos Caro P.M.C., Klippstein R., Pozo D., Zaderenko A.P. (2010). Silver Nanoparticles: Sensing and Imaging Applications. Silver Nanoparticles.

[50] Wang M., Ma X., Zong S., Su Y., Su R., Zhang H., Liu Y., Wang C., Li Y. (2024). The prescription design and key properties of nasal gel for CNS drug delivery: A review. Eur. J. Pharm. Sci..

[51] Lefrançois P., Ibarboure E., Payré B., Gontier E., Le Meins J.-F., Schatz C. (2015). Insights into Carbopol gel formulations: Microscopy analysis of the microstructure and the influence of polyol additives. J. Appl. Polym. Sci..

[52] Khaliq N.U., Lee J., Kim S., Sung D., Kim H. (2023). Pluronic F-68 and F-127 Based Nanomedicines for Advancing Combination Cancer Therapy. Pharmaceutics.

[53] Lund M.E., Hawkinson R.W. (1983). Evaluation of the Prompt inoculation system for preparation of standardized bacterial inocula. J. Clin. Microbiol..

[54] Chung E., Ren G., Johnston I., Matharu R.K., Ciric L., Walecka A., Cheong Y.-K. (2023). Applied Methods to Assess the Antimicrobial Activity of Metallic-Based Nanoparticles. Bioengineering.

[55] Hosseini R.S., Hasanpour K., Khoshnevis M., Fakhr M.S., Derin E., Ghaffarian A., Kement C. (2024). Therapeutic Effect of Silver Nanoparticles in the Management of Diabetic Ulcers: A Systematic Review and Meta-Analysis on RCTs. Int. J. Low. Extrem. Wounds.

[56] de Souza A., Vora A.H., Mehta A.D., Moeller K., Moeller C., Willoughby A.J.M., Godse C.S., Kumar P., Kothari V. (2021). SilverSol^®^ a Nano-Silver Preparation: A Multidimensional Approach to Advanced Wound Healing. Wound Healing Research: Current Trends and Future Directions.

[57] Ranoszek-Soliwoda K., Tomaszewska E., Socha E., Krzyczmonik P., Ignaczak A., Orlowski P., Krzyzowska M., Celichowski G., Grobelny J. (2017). The role of tannic acid and sodium citrate in the synthesis of silver nanoparticles. J. Nanoparticle Res..

[58] McClary F.A., Gaye-Campbell S., Hai Ting A.Y., Mitchell J.W. (2013). Enhanced localized surface plasmon resonance dependence of silver nanoparticles on the stoichiometric ratio of citrate stabilizers. J. Nanoparticle Res..

[59] Gorham J.M., Rohlfing A.B., Lippa K.A., MacCuspie R.I., Hemmati A., David Holbrook R. (2014). Storage Wars: How citrate-capped silver nanoparticle suspensions are affected by not-so-trivial decisions. J. Nanoparticle Res..

[60] Valenti L.E., Giacomelli C.E. (2017). Stability of silver nanoparticles: Agglomeration and oxidation in biological relevant conditions. J. Nanoparticle Res..

[61] Heuer-Jungemann A., Feliu N., Bakaimi I., Hamaly M., Alkilany A., Chakraborty I., Masood A., Casula M.F., Kostopoulou A., Oh E. (2019). The Role of Ligands in the Chemical Synthesis and Applications of Inorganic Nanoparticles. Chem. Rev..

[62] Bélteky P., Rónavári A., Igaz N., Szerencsés B., Tóth I.Y., Pfeiffer I., Kiricsi M., Kónya Z. (2019). Silver nanoparticles: Aggregation behavior in biorelevant conditions and its impact on biological activity. Int. J. Nanomed..

[63] Franco-Ulloa S., Tatulli G., Bore S.L., Moglianetti M., Pompa P.P., Cascella M., De Vivo M. (2020). Dispersion state phase diagram of citrate-coated metallic nanoparticles in saline solutions. Nat. Commun..

[64] Duval R.E., Gouyau J., Lamouroux E. (2019). Limitations of Recent Studies Dealing with the Antibacterial Properties of Silver Nanoparticles: Fact and Opinion. Nanomaterials.

[65] Vazquez-Muñoz R., Bogdanchikova N., Huerta-Saquero A. (2020). Beyond the Nanomaterials Approach: Influence of Culture Conditions on the Stability and Antimicrobial Activity of Silver Nanoparticles. ACS Omega.

[66] Gimenez-Ingalaturre A.C., Rubio E., Chueca P., Laborda F., Goñi P. (2022). Contribution to optimization and standardization of antibacterial assays with silver nanoparticles: The culture medium and their aggregation. J. Microbiol. Methods.

[67] Sharma S., Bose A., Biswas S., Sen S., Roy I. (2025). Cyperus rotundus mediated green synthesis of silver nanoparticles for antibacterial wound dressing applications. Sci. Rep..

[68] Russo E., Villa C. (2019). Poloxamer Hydrogels for Biomedical Applications. Pharmaceutics.

[69] Kerkhofs S., Willhammar T., Van Den Noortgate H., Kirschhock C.E.A., Breynaert E., Van Tendeloo G., Bals S., Martens J.A. (2015). Self-Assembly of Pluronic F127—Silica Spherical Core–Shell Nanoparticles in Cubic Close-Packed Structures. Chem. Mater..

[70] Sarfraz M., Iqbal R., Khan K.U., Minhas M.U. (2022). Carbopol Based Hydrogels for ITOPRIDE Hydrochloride Delivery; Synthesis, Characterization and Comparative Assessment with Various Monomers. J. Funct. Biomater..

[71] Liu T., Aman A., Ainiwaer M., Ding L., Zhang F., Hu Q., Song Y., Ni Y., Tang X. (2021). Evaluation of the anti-biofilm effect of poloxamer-based thermoreversible gel of silver nanoparticles as a potential medication for root canal therapy. Sci. Rep..

[72] Marta B., Jakab E., Potara M., Simon T., Imre-Lucaci F., Barbu-Tudoran L., Popescu O., Astilean S. (2014). Pluronic-coated silver nanoprisms: Synthesis, characterization and their antibacterial activity. Colloids Surf. A Physicochem. Eng. Asp..

[73] Ganguly R., Kumar S., Tripathi A., Basu M., Verma G., Sarma H.D., Chaudhari D.P., Aswal V.K., Melo J.S. (2020). Structural and therapeutic properties of Pluronic^®^ P123/F127 micellar systems and their modulation by salt and essential oil. J. Mol. Liq..

[74] Skwarczynski M., Bashiri S., Yuan Y., Ziora Z.M., Nabil O., Masuda K., Khongkow M., Rimsueb N., Cabral H., Ruktanonchai U. (2022). Antimicrobial Activity Enhancers: Towards Smart Delivery of Antimicrobial Agents. Antibiotics.

[75] Dakal T.C., Kumar A., Majumdar R.S., Yadav V. (2016). Mechanistic Basis of Antimicrobial Actions of Silver Nanoparticles. Front. Microbiol..

[76] Bruna T., Maldonado-Bravo F., Jara P., Caro N. (2021). Silver Nanoparticles and Their Antibacterial Applications. Int. J. Mol. Sci..

[77] Qing Y., Cheng L., Li R., Liu G., Zhang Y., Tang X., Wang J., Liu H., Qin Y. (2018). Potential antibacterial mechanism of silver nanoparticles and the optimization of orthopedic implants by advanced modification technologies. Int. J. Nanomed..

[78] Alvarado-Gomez E., Martínez-Castañon G., Sanchez-Sanchez R., Ganem-Rondero A., Yacaman M.J., Martinez-Gutierrez F. (2018). Evaluation of anti-biofilm and cytotoxic effect of a gel formulation with Pluronic F-127 and silver nanoparticles as a potential treatment for skin wounds. Mater. Sci. Eng. C.

[79] Prusty A., Parida P. (2015). Development and Evaluation of Gel Incorporated with Biogenically Synthesised Silver Nanoparticles. J. Appl. Biopharm. Pharmacokinet..

[80] Ershov V.A., Ershov B.G. (2024). Effect of Silver Nanoparticle Size on Antibacterial Activity. Toxics.

[81] Tănase M.A., Soare A.C., Diţu L.M., Nistor C.L., Mihaescu C.I., Gifu I.C., Petcu C., Cinteza L.O. (2022). Influence of the Hydrophobicity of Pluronic Micelles Encapsulating Curcumin on the Membrane Permeability and Enhancement of Photoinduced Antibacterial Activity. Pharmaceutics.

